# Deep Graph Learning for Circuit Deobfuscation

**DOI:** 10.3389/fdata.2021.608286

**Published:** 2021-05-24

**Authors:** Zhiqian Chen, Lei Zhang, Gaurav Kolhe, Hadi Mardani Kamali, Setareh Rafatirad, Sai Manoj Pudukotai Dinakarrao, Houman Homayoun, Chang-Tien Lu, Liang Zhao

**Affiliations:** ^1^Department of Computer Science and Engineering, Mississippi State University, Starkville, MS, United States; ^2^Department of Computer Science, Virginia Tech, Falls Church, VA, United States; ^3^Electrical and Computer Engineering Department, University of California, Davis, Davis, CA, United States; ^4^Department of Electrical and Computer Engineering, George Mason University, Fairfax, VA, United States; ^5^Computer Science, University of California, Davis, Davis, CA, United States; ^6^Department of Computer Science, Emory University, Atlanta, GA, United States

**Keywords:** graph mining, circuit deobfuscation, satisfiability checking, graph neural networks, deep learning

## Abstract

Circuit obfuscation is a recently proposed defense mechanism to protect the intellectual property (IP) of digital integrated circuits (ICs) from reverse engineering. There have been effective schemes, such as satisfiability (SAT)-checking based attacks that can potentially decrypt obfuscated circuits, which is called deobfuscation. Deobfuscation runtime could be days or years, depending on the layouts of the obfuscated ICs. Hence, accurately pre-estimating the deobfuscation runtime within a reasonable amount of time is crucial for IC designers to optimize their defense. However, it is challenging due to (1) the complexity of graph-structured circuit; (2) the varying-size topology of obfuscated circuits; (3) requirement on efficiency for deobfuscation method. This study proposes a framework that predicts the deobfuscation runtime based on graph deep learning techniques to address the challenges mentioned above. A conjunctive normal form (CNF) bipartite graph is utilized to characterize the complexity of this SAT problem by analyzing the SAT attack method. Multi-order information of the graph matrix is designed to identify the essential features and reduce the computational cost. To overcome the difficulty in capturing the dynamic size of the CNF graph, an energy-based kernel is proposed to aggregate dynamic features into an identical vector space. Then, we designed a framework, Deep Survival Analysis with Graph (DSAG), which integrates energy-based layers and predicts runtime inspired by censored regression in survival analysis. Integrating uncensored data with censored data, the proposed model improves the standard regression significantly. DSAG is an end-to-end framework that can automatically extract the determinant features for deobfuscation runtime. Extensive experiments on benchmarks demonstrate its effectiveness and efficiency.

## 1. Introduction

**Figure 1:**
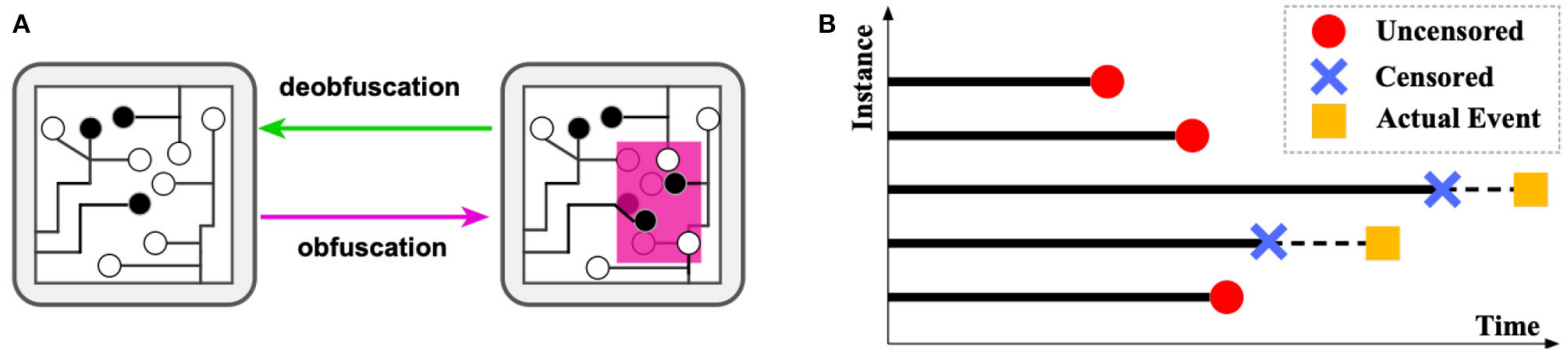
**(A)** An illustration of obfuscation and deobfuscation. **(B)** An illustration demonstrating the survival analysis problem.

The considerable high capital costs on semiconductor manufacturing motivate most hi-tech companies to outsource their designed integrated circuits (ICs) for fabrication. Despite the reduced cost and other benefits, this trend has led to ever-increasing security risks, such as concerns of risks on IC counterfeiting, piracy, and unauthorized overproduction by the contract foundries (Subramanyan et al., 2015). The overall financial risk caused by such counterfeit and unauthorized ICs was estimated to be over $169 billion per year (Informa.com, 2012). The major threats from attackers arise from reverse engineering an IC by fully identifying its functionality layer-by-layer and extracting the unveiling gate-level netlist. To prevent such reverse engineering, IC *obfuscation* techniques have been extensively researched in recent years (Yasin et al., 2017). The general idea is to obfuscate some gates in an IC so that their gate type cannot be determined by reverse engineering optically, yet they preserve the same functionality as the original gates. As shown in Figure 1A, obfuscation is a process that selects a part of the circuit (in pink) and modifies the structure, whose functionality can be retrieved only if correct keys are provided at the additional input gates.

Such techniques were highly effective until recently, when attacking techniques based on logical attackers were invented and widely applied (El Massad et al., 2015). More recently, efficient methods, such as satisfiability (SAT)-checking based attacks have been proposed, which have attracted enormous attention (Liu et al., 2016). The runtime of reverse engineering by SAT attack on the IC mostly depends on the complexity of the obfuscated IC, which can vary from milliseconds to days or years. Therefore, a successful obfuscation requires a prohibitive amount of time (i.e., many years) for attackers to deobfuscate. However, gates to obfuscate come at a high cost in finance, power, and space; such trade-off forces us to search for an optimal layout instead of purely increasing their quantity. Therefore, the best set of gates for obfuscating maximizes the runtime for deobfuscating. However, until present, selecting the best set of gates is still generally based on human heuristics or experience, which is seriously arbitrary and suboptimal (Khaleghi and Rao, 2018). This is due to the inability to “try and error” all the different ways of obfuscation, as there are millions of combinations to try, and the runtime for each try (i.e., to run the attacker) can be extremely time-consuming. This situation also causes the early stop of numerous attack simulations since the unknown attack times may be dramatically long, and the computational resource is limited. Such incomplete simulations cannot be used by normal regression models due to their inaccurate labels (i.e., early stop time). These incomplete records are similar to censored data in survival analysis (Wang et al., 2019), as shown in Figure 1B. The actual event of censored data (i.e., the actual time of finishing deobfuscation) is unknown; however, the censored time point is recorded. For uncensored data, the recorded time is the exact time taken for completing deobfuscation.

This research topic is vastly underexplored because of its significant challenges: **(1) Difficulty in characterizing the hidden and sophisticated algorithmic mechanism of attackers**. Over the recent years, a large number of deobfuscation methods have been proposed with various techniques (Khaleghi and Rao, 2018). In order to practically beat the attackers, methods with sophisticated theories, rules, and heuristics have been proposed and adopted. The behavior of such highly non-linear and strongly coupled systems is prohibitive for conventional simple models [e.g., linear regression and support vector machine (Bishop and Mitchell, 2006)] to characterize. **(2) Difficulty in extracting determinant features from discrete and dynamic graph-structured ICs**. The inputs of the runtime estimation problem are the ICs with selected obfuscated gates. Conventional feature extraction methods are not intuitively applied to such type of varying-structured data without significant information loss. Hence, it is highly challenging to intactly formulate and seamlessly integrate them as mathematical forms that can be input to conventional machine learning models. **(3) Requirement on high efficiency for deobfuscation runtime estimation**. The key to the defense against deobfuscation is speed. The faster the defender can estimate the deobfuscation runtime for each candidate set of obfuscated gates, the more candidate sets the defender can estimate, hence, the better the obfuscation effect will be. Moreover, the estimation speed of deobfuscation runtime must not be sensitive to different obfuscation strategies in order to make the defender strategy controllable.

This study addresses all the above challenges and proposes the first generic framework for deobfuscation runtime prediction, based on conjunctive normal form (CNF) graph representation for obfuscated circuits. The major contributions of this paper are:

- **Formulating a graph learning framework with survival analysis for predicting deobfuscation runtime**. In the proposed method, a graph-based model is built by transforming obfuscated ICs into a CNF graph. The model then learns the relationship between the complexity of the CNF graph and deobfuscation runtime. By introducing survival analysis, the model can integrate complete simulations with incomplete simulations, significantly improving the prediction performance.
- **Proposing a feature extraction method for deobfuscation runtime estimation based on graph deep learning**. To model SAT-based deobfuscation, a multi-order CNF graph is proposed to derive informative representations from obfuscated ICs. Such an end-to-end deep graph regressor can automatically extract the discriminative features that are determinants to estimate the deobfuscation runtime to achieve accurate runtime prediction.
- **Designing an energy-based neural layer to process varying-size of graph data**. To unify the dynamic topology of the CNF graph, this study innovatively leveraged the energy of restricted Boltzmann machines to quantify the complexity of CNF. The bipartivity of the CNF graph is highly utilized to optimize the computational cost.
- **Conducting comprehensive experimental evaluations and analyses on multiple datasets**. The proposed method is compared with several state-of-the-art methods on three benchmark datasets. The analyses of the performance and effectiveness demonstrated the advantage of our method.

## 2. Problem Setup

Mathematically, solving the IC deobfuscation problem is often considered equivalent to solving the Boolean SAT of a CNF (Yasin et al., 2016a; Shamsi et al., 2017; Zhou et al., 2017; Roshanisefat et al., 2018; Xie and Srivastava, 2018; Zamiri Azar et al., 2018). Specifically, the obfuscated IC, where several gates have been encrypted by replacing old gates with new gates and adding key inputs, can be equivalent to the original IC only when the key inputs are correctly inferred. Such a problem is routinely formulated as a CNF expression, and solving this problem is equivalent to solving a standard SAT problem, which has been proved to be NP-complete (Cook, 1971; Karp, 1972). This means that the solving runtime of obfuscated IC cannot be tightly estimated theoretically, and hence, an accurate runtime prediction method is imperative for obfuscation assessment. Therefore, the runtime prediction is dependent on the CNF expression because it stores all the necessary information for runtime estimation, as mentioned above. As a standard formula of a Boolean expression, CNF is a conjunction of one or more clauses, where a clause is a disjunction of variables. In other words, in CNF, different clauses are connected by “AND” operators, while each clause consists of one or more variables (or their negations) connected by “OR” operators. In practice, the CNF formation of an obfuscated IC can be conventionally generated through a set of handwritten rules (Subramanyan et al., 2015).

The above is a new yet extremely challenging research problem that involves three major technical challenges: **(1) Difficulty in representing CNF in an intact and structured way for a machine learning model**. CNF, though typically written as a sequence, is mathematically not a sequence as the order among different clauses is meaningless. Moreover, one variable can appear in multiple clauses with or without their negation forms, which further complicates its representation. However, there is no such existing machine learning technique that is designed for directly modeling varying-size CNF expression; however, extracting handcrafted features from the technique will surely cause loss of information and would be easily biased. **(2) Difficulty in learning the mapping from the CNF to the runtime**. Different from conventional inputs of machine learning models, CNF inherently endorses logical operators (typically discrete) instead of numerical operators. Moreover, it is imperative, yet challenging, to automatically learn the determinant features that decide how “time-consuming” deobfuscating a CNF is.

**Problem Formulation:** Given the CNF (denoted as Γ_*i*_) of the *i*th SAT instance, the goal of this study is **(1) Accuracy:** to predict the runtime by a prediction function, $f_{pred}:\Gamma_{i}\to T_{i}\in {\mathbb{R}}^{+}$, where *T*_*i*_ is runtime of an SAT solver on the SAT instance Γ_*i*_; **(2) Efficiency** *t*_*i*_ ≪ *T*_*i*_, where *t*_*i*_ is the time consumption of *f*_*pred*_. This means that prediction should be much faster than the real runtime. Otherwise, there is no superiority beyond directly running an SAT solver.

## 3. Deep Survival Analysis for CNF Graph

To address the above challenges, we first present a comprehensive representation of the CNF graph representation and then present an energy model for feature aggregation. Last, we elaborate survival analysis with the proposed graph representation learning.

**Figure 2:**
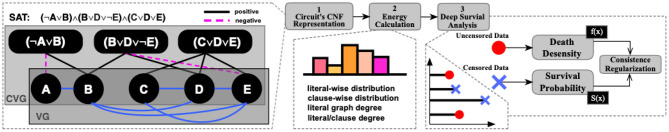
Architecture of Deep Survival Analysis with Graph (DSAG): **(1)** extract Variable Graph/Clause-Variable Graph (VG/CVG) based on conjunctive normal form (CNF) formula; **(2)** apply energy kernel to aggregate dynamic-size features; **(3)** employ survival analysis to exploit censored data with uncensored data.

As shown in the first component in Figure 2, a CNF-SAT instance is represented in two graph formats, namely *VG* (variable graph) and *CVG* (clause-variable graph), which will be introduced in “CNF Graph Representation of IC” section. Then, multi-order information is generalized from these graphs by calculating the power series of its adjacency matrix. To minimize the computational cost, we leverage the bipartivity property among the graphs. After extracting a set of intermediate features from graph representations, an energy-based kernel is proposed to model the dynamic-size data. Finally, survival analysis is leveraged to improve the runtime estimation of deobfuscation.

### 3.1. CNF Graph Representation of IC

It is clear that modern SAT solvers exploit the structure of CNF (Ansótegui and Levy, 2011; Ansótegui et al., 2012; Newsham et al., 2014; Giráldez-Cru and Levy, 2015; Mull et al., 2016). Unlike the previous existing studies, this study explores this hidden pattern by applying graph neural networks rather than using handcrafted graph features based on the domain knowledge. Given a CNF instance, we employ two graphs:

**Definition 3.1**. ***Clause-Variable Graph (CVG)** Given a SAT instance, Γ, over the set of variables, *X*, and clause, *c*, its clause-variable graph is a undirected and signed graph G(V,E), where V∈X∪c, and the value of $E_{ij}$ is defined as: **(a)** 1 (positively connected), if *X*_*i*_ is in *c*_*j*_, and *X*_*i*_ is positive; **(b)** −1 (negatively connected), if *X*_*i*_ is in *c*_*j*_, and *X*_*i*_ is negative; **(c)** 0 (disconnected), if *X*_*i*_ is not in *c*_*j*_*.

**Definition 3.2**. ***Variable Graph (VG)** Given a SAT instance, Γ, over the set of variables, *X*, its variable graph is a graph $G\left(V^{\prime },E^{\prime }\right)$, where $V^{\prime }\in X$, and the value of $E_{ij}^{\prime }$ is defined as the count of clauses in which both *X*_*i*_ and *X*_*j*_ exist*.

Since there exist positive and negative entries in the connectivity of CVG, our model distinguishes the two groups by characterizing them separately. Specifically, CVG is divided into two groups: CVG^+^ keeps all values that are 1, while CVG^−^ keeps all values that are −1, and then, −1 entry has been set to 1 for convenience. To make the notation succinct in the rest of this study, CVG includes two matrices (CVG^+^, CVG^−^), and $A=A_{CVG}$. Typically, the CVG/VG for different instances are different, leading to VG_*m*_ ≠ VG_*n*_, and CVG_*m*_ ≠ CVG_*n*_, given *m* ≠ *n*.

**Lemma 3.1**. *The adjacency matrix of VG, $A_{VG}$, can be obtained from raising the adjacency matrix of CVG to the second power*.

*Proof*. A) is the normal adjacency matrix of CVG. We define a matrix: $A^{2}=|A|\cdot |A|$. Therefore, the entry at i-th row and j-th column of $A^{2}$ is:

(1) $$a_{i,j}^{\left(2\right)}=\sum_{k=1}^{N}\left|a_{i,k}^{\left(1\right)}\right|\;\cdot |a_{k,j}^{\left(1\right)}|\;=\{\begin{array}{ll} 1 & \text{ if }a_{i,j}^{\left(2\right)}>0\\ 0 & \text{ if }a_{i,j}^{\left(2\right)}=0 \end{array}$$

where *a*^(1)^ and *a*^(2)^ denote the entry in A and $A^{2}$, respectively, [$a_{i,j}^{\left(n\right)}=A^{n}\left(i,j\right)$], and *a*^(1)^ ∈ {0, 1}. Therefore, if there exist a path from node *i* to *k* (i.e., $a_{i,k}^{\left(1\right)}=1$) and a path from *k* to *j* (i.e., $a_{k,j}^{\left(1\right)}=1$), then count one more value for $a_{i,j}^{\left(2\right)}\leftarrow a_{i,j}^{\left(2\right)}+1$. Note that *a* could indicate literal-literal, literal-clause, or clause-clause relationship. Following definition 3.2, if literal *i* and *j* share |*C*_*i, j*_| clauses in CVG, count |*C*_*i,j*_| for the entry $A_{VG}\left(i,j\right)$. Therefore, $A_{VG}$ is included in $A^{2}$ when *a* is literal-literal. Specifically, it can be written as:

$$\begin{array}{l} A^{2}=\left[\begin{array}{cc} A_{VG} & 0\\ 0 & A_{CG} \end{array}\right], \end{array}$$

where $A_{CG}$ is clause-to-clause matrix which has a similar definition as Equation (1).

**Extend to higher power:** Proof of Lemma 3.1 is provided in the support material. This Lemma means that VG can be mathematically obtained from raising the power of CVG to 2, i.e., $A^{2}$. Note that the adjacency of VG is not equal to $A^{2}$, but contained in $A^{2}$. Therefore, the first (CVG) and second (VG) power of A represent different physical meanings, and CVG covers VG by raising its power. Similarly, higher powers of A also derive determinant information. Suppose we have Γ = (*x*_1_ ∨ *x*_2_) ∧ (¬*x*_1_ ∨ *x*_3_), and *c*_1_ = (*x*_1_ ∨ *x*_2_), *c*_2_ = (¬*x*_1_ ∨ *x*_3_). Therefore, *x*_2_ and *c*_2_ are third order neighbors in CVG, and *x*_2_ and *x*_3_ are fourth order neighbors in CVG. To make this Γ true, both *x*_2_ and *x*_3_ must be true. *x*_1_ is not useful and no matter takes 1 or 0. Therefore, the constraint from the third and fourth order information also reveals critical inference for solving the SAT problem. This inspires the utilization of multiple powers of A as input.

### 3.2. Energy Model for CNF Graph

Unlike the conventional graphs, the correlation among the neighboring nodes in the CNF graph does not indicate “proximity” or “similarity,” but instead it indicates logical relation with signed edges. Moreover, almost each CNF graph is with a different node size. To address those issues, a novel graph encoder layers have been proposed by leveraging and extending the energy of restricted Boltzmann machines (RBMs) (Nair and Hinton, 2010). By innovatively treating variables and clauses as visible and hidden units, CNF graphs can be modeled by RBMs.

Most existing graph deep learning operators focus on graphs with fixed topology, while the size and topology of CNF graph vary across different instances. To solve this problem, we design a kernel for aggregating interaction information in one graph. Specifically, a *d*-dimensional vector of pseudo-coordinates is associated with [*v, h*], which denotes variable and clause, respectively. We define a weighting kernel *Z*_Θ_(·, ·), so that, for one CNF bipartite graph $G_{i}$, we have:

(2) $$\begin{array}{l} E_{interaction}=\underset{m}{\sum }\underset{n}{\sum }Z_{\Theta }\left(E\left(v_{m},h_{n}\right)\right)\cdot E\left(v_{m},h_{n}\right), \end{array}$$

where mapping function *Z*_Θ_(·) projects the edge of variable-clause pair $E\left(v_{m},h_{n}\right)$ into a new value as the weight, i.e., $Z_{\Theta }\left(E\left(v_{m},h_{n}\right)\right)$. E is an energy function that the model will learn. Note that *Z*_Θ_ function is implemented by neural networks and controlled by fixed-size parameters Θ. Similarly, we further generalize *E*_*variable*_, *E*_*clause*_ as $E_{variable}=\underset{m}{\sum }Z_{\Theta }\left(v_{m}\right)\cdot v_{m},$ and $E_{clause}=\underset{n}{\sum }Z_{\Theta }\left(h_{n}\right)\cdot h_{n}$, where *v* and *h* indicate degree distribution of variable and clause, respectively. Clause and variable are also important, in that the ratio of clause over variable w.r.t. degree sum exhibits significant phase transit phenomenon, which is often widely recognized in CNF structure (Friedrich and Rothenberger, 2018). Therefore, the deep energy model for CNF graph (EnCNF) is:

(3) $$\begin{array}{l} \begin{array}{c} E=f(\overset{variable}{\overset{︷}{\underset{m}{\sum }Z_{\Theta }\left(v_{m}\right)\cdot v_{m}}},\overset{clause}{\overset{︷}{\underset{n}{\sum }Z_{\Theta }\left(h_{n}\right)\cdot h_{n}}},\\ \overset{interaction}{\overset{︷}{\underset{m}{\sum }\underset{n}{\sum }Z_{\Theta }\left(E\left(v_{m},h_{n}\right)\right)\cdot E\left(v_{m},h_{n}\right)}},) \end{array} \end{array}$$

Equation (3) above does not consider the sign of the edges between variable and clauses. Hence, positive and negative entries are encoded separated, i.e., *E* = {*E*^+^, *E*^−^}.

The following analysis provides the relationship analysis between EnCNF and an existing GNN. As a state-of-art of graph neural networks model, the superiority of DCNN (Atwood and Towsley, 2016) beyond the other graph deep learning models is the capacity in handling graphs with variable sizes and considering multiple orders, which is also one advantage of our model. The study demonstrates that DCNN is a special case of our EnCNF:

**Lemma 3.2**. *EnCNF is a generalization of DCNN, while DCNN is a special case of EnCNF when setting the feature aggregation to *mean* function*.

*Proof*. DCNN can be extended to whole graph modeling by taking the mean activation over the features on diffusion $P_{t}^{*}X_{t}$:

(4) $$\begin{array}{l} Z=f\left(W^{c}⊙1_{N_{t}}^{⊤}P_{t}^{*}X_{t}/N_{t}\right)=f\left(W^{c}⊙\underset{\text{aggregation}}{\underset{︸}{{\left(\frac{1}{N_{t}}\right)}_{N_{t}}^{⊤}}}P_{t}^{*}X_{t}\right), \end{array}$$

where ${\left(\frac{1}{N_{t}}\right)}_{N_{t}}$ is a *N*_*t*_ × 1 vector of value $\left(\frac{1}{N_{t}}\right)$, *t* indicates the index of graph instance, *W*^*c*^ is a real-valued weight tensor, and P^*^ is power series of adjacency matrix. ⊙ and ⊤ are element-wise multiplication and matrix transpose, respectively. Following the same representation, we can rewrite DSAG as:

(5) $$\begin{array}{l} Z=f\left(W^{c}⊙\underset{\text{aggregation}}{\underset{︸}{f_{E}\left(\cdot \right)}}P_{t}^{*}X_{t}\right), \end{array}$$

where *f*_*E*_(·) represents a vector of ${\left[Z_{\Theta }(\phi (i))\right]}_{i=0}^{d_{feat}-1}$, and *d*_*feat*_ indicate dimension of a feature and ϕ(*i*) is the i-th value along a feature. Therefore, *f*_*E*_(·) is a vector of dynamic size.

**Figure 3:**
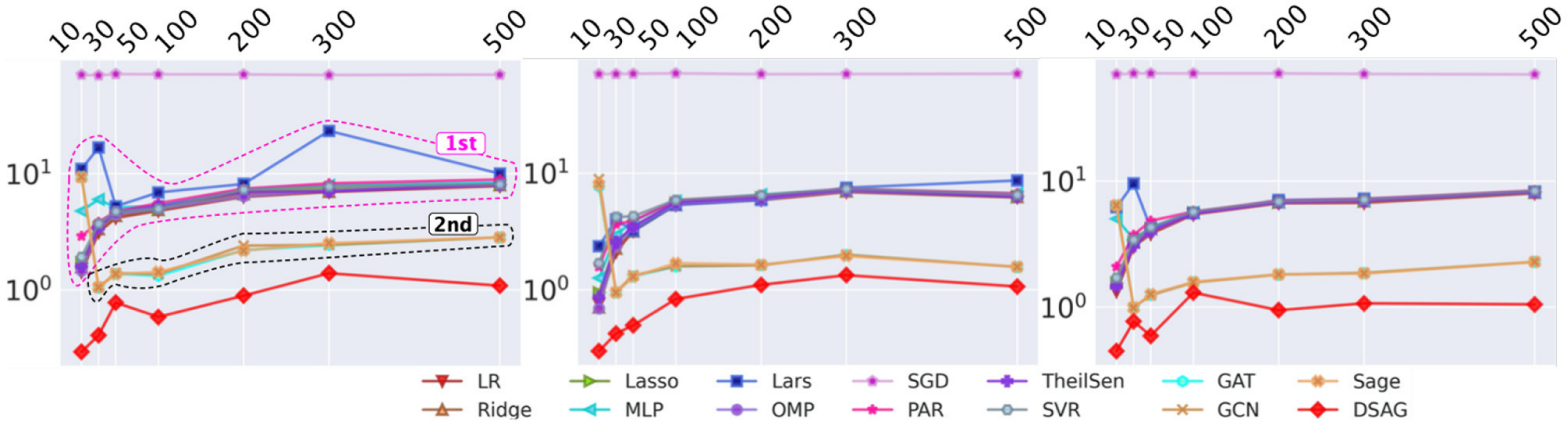
Comparison with DCNN.

As shown in Figure 3, *f*_*E*_(·) calculate a weight vector for each number of feature maps by repeatedly applying the one-dimensional function *Z*_Θ_·. This difference brings two advantages: (1) The feature aggregation is learned from the data rather than average, which is extremely important for the high non-linearity in graph topology; (2) DSAG can work for the case where the dimension of feature changes across different instances, while DCNN still requires the dimension to be fixed.

### 3.3. RBM Model for Extracting Signed Information

#### 3.3.1. Energy Model for 1st-Order Graph Operators

To capture the sign information, the corresponding incidence matrix $M\in {\mathbb{R}}^{\left|V^{variable}|\times |V^{clause}\right|}$ is utilized:

(1) Normalized positive and negative edge distribution in clause scope (**NPNC**): we count positive and negative edges for each clause and take normalization of both positive and negative counts, so there are two values for each clause, i.e., *c*^*pos*^ and *c*^*neg*^. If there exist |*V*^*clause*^| clauses, there will be two |*V*^*clause*^| features:

(6) $$\langle c_{clause}^{+}(0),c_{clause}^{-}(0)\rangle ,\langle c_{clause}^{+}(1),c_{clause}^{-}(1)\rangle ,...,\langle c_{clause}^{+}(|V^{clause}|-1),c_{clause}^{-}(|V^{clause}|-1)\rangle ,$$

(2) Normalized positive and negative edge distribution in variable scope (**NPNL**): Similarly, positive and negative degrees are counted for each variable, and normalization per variable is taken. There will be two |*V*^*variable*^| features:

(7) $$\begin{array}{l} \langle c_{variable}^{+}(0),c_{variable}^{-}(0)\rangle ,\langle c_{variable}^{+}(1),c_{variable}^{-}(1)\rangle ,...,\langle c_{variable}^{+}(|V^{variable}|-1),\\ \;c_{variable}^{-}(|V^{variable}|-1)\rangle . \end{array}$$

Equations (6) and (7) then are fed as features into Equation (2).

#### 3.3.2. Energy Model for the 2nd-Order Graph Operators

The 2nd power of A with adjacency matrix $A^{2}$ denotes the variable-wise and clause-wise mutual information, which corresponds to non-zero blocks as shown in the upper matrix of the 2nd step in Figure 2. Their physical meaning includes (1) **variable-wise** frequency of two variables appearing in the same clause and (2) **clause-wise** frequency of two clauses sharing the same variable. Intuitively, whether two variables share the same clause or not is more important than how many times they share. To further emphasize this important trait and reduce the computational complexity, our model only distinguish zero with non-zero entries in 2nd power of A, which means that we only consider if two variables co-appear in the same clause or two clauses share the same variable at least one time. This can be obtained by setting all non-zero value of $A^{2}$ to 1. Therefore, in VG graph (first component of Figure 2), there exists a positive link (in blue) only, but the original adjacency of $A^{2}$ (the second component of Figure 2) may have both positive and negative entries. Finally, Equation (3) is applied to calculate the energy of this graph, with first and second order of graph information.

### 3.4. Deep Survival Analysis

Due to the difficulty of obtaining labeled instance with large runtime (i.e., hard instance), it may take months. Therefore, there exist numerous records that stop early, and their exact runtime is unknown, which will be referred to as “censored” data in this context. Censored data cannot be provided as labels for the regression model, but they still offer information about runtime. For example, if a simulation stops within 1 h, the real runtime is probably larger than 1 h.

Survival analysis was introduced to integrate uncensored data with censored data, improving the performance of regression performance. Specifically, we borrowed the idea of parametric survival analysis (Li et al., 2016) that can be used to predict the time. It finds the best parameters by optimizing the likelihood function of all instances:

(8) $$\begin{array}{l} L\left(\beta \right)=\overset{uncensored}{\overset{︷}{\underset{\delta_{i}=0}{\prod }f\left(T_{i},\beta \right)}}\overset{censored}{\overset{︷}{\underset{\delta_{i}=1}{\prod }S\left(T_{i},\beta \right)}}, \end{array}$$

where δ_*i*_ = 0 and δ_*i*_ = 1 mean uncensored and censored data, *T*_*i*_ denotes predicted time, and β is the model parameter. Note that taking logarithm on Equation (8) will convert the product into sum function, i.e., $\log L\left(\beta \right)=\underset{\delta_{i}=0}{\sum }\log f\left(T_{i},\beta \right)+\underset{\delta_{i}=1}{\sum }\log S\left(T_{i},\beta \right)$. *f*(*t*), *S*(*t*) indicate death density function and survival function, respectively (Wang et al., 2019). Under survival analysis, *f*(*t*) is defined as the probability that the instance dies at time t, while *S*(*t*) indicates the probability that the instance can survive for longer than a certain time t. However, Equation (8) is designed for the parametric model, in which *f*(*t*) and *S*(*t*) have analytical forms, which is difficult to be determined, especially in a complex and real-world scenario.

Inspired by these concepts, we designed a new objective function, imposing similar regularization to Equation (8). To further improve the accuracy of prediction, a consistence loss is added to reconcile uncensored loss and censored loss. Therefore, the proposed loss consists of three components with weight parameters α and β:

(9) $$\begin{array}{l} L=L_{uncensored}+\alpha L_{censored}+\beta L_{consist}. \end{array}$$

**Uncensored Loss** is designed to represent the regression loss for the uncensored data. We define *f*_*reg*_(*x*) as regression model and the uncensored loss is written as:

(10) $$\begin{array}{l} L_{uncensored}=\underset{\delta =0}{\sum }||f_{reg}\left(X\right)-\log Y|{|}^{2}, \end{array}$$

where *X* is the features of instance, and *Y* is the labeled runtime of uncensored data (i.e., exact runtime). Both death density function and survival function are exponential based due to the death-age relationship. The exponential base is also employed in *f*_*reg*_, that is why our target is log*Y* rather than *Y*. **Censored Loss** characterizes the capacity that distinguishes whether an instance exceed a censor threshold or not. Utilizing both censored and uncensored data, a binary classification task is defined (i.e., larger or smaller than the threshold). Therefore, its binary cross-entropy loss is:

(11) $$\begin{array}{l} L_{censored}=-\underset{\delta \in \left\{0,1\right\}}{\sum }\delta \log f_{class}\left(X\right), \end{array}$$

where *f*_*class*_(*x*) is a binary classifier. Equation (10) is called uncensored loss since only uncensored data is considered. Equation (11) is termed as censored loss since it is designed to learn whether an instance is below or beyond the threshold.

Note that uncensored loss and censored loss correspond to those two components in Equation (8). However, there is a implicit connection between *f*(*t*) and *S*(*t*) (Wang et al., 2019):

(12) $$\begin{array}{l} f\left(t\right)=\left(1-S\left(t\right)\right)t=-S\left(t\right)t, \end{array}$$

and they share the same parameter β. Therefore, a regularization is proposed to implement this mechanism between *f*_*ref*_ and *f*_*class*_. **Consistence Loss** is a constraint that forces *f*_*reg*_ to predict the right label as *f*_*class*_ does. Intuitively, if an instance is censored, *f*_*reg*_ must provide the value that is large than the threshold as output even though the exact runtime is unknown for the censored instance. Specifically, consistence loss is defined as a ReLU function:

(13) $$\begin{array}{l} L_{consist}=ReLU\left[\left(f_{reg}-\Gamma \right)\cdot \left(1-2\delta \right)\right], \end{array}$$

where Γ is a fixed censor threshold, and (1−2δ) is a mapping that transfers δ ∈ {0, 1} to δ ∈ {1, −1}. Therefore, when *f*_*reg*_ > Γ (i.e., the test instance is hard instance, and should be censored) and δ = 0 (i.e., uncensored), it is inconsistent. In this case, we add a punishment as above: (*f*_*reg*_ − Γ) and (1 − 2δ) always have the same sign if inconsistence happens. ReLU is employed to remove consistent cases from loss.

## 4. Background and Related Work

### 4.1. Logic Obfuscation and SAT Attacks

Logic obfuscation, often referred to as logic locking (Yasin et al., 2016b), is a hardware security solution that facilitates hiding the Intellectual Property (IP) using key-programmable logic gates. Although obfuscation schemes try to minimize the probability of determining the correct key by an attacker and avoid making pirated and illegal copies, introducing SAT attack shows that these schemes can be broken (Subramanyan et al., 2015). In order to perform the SAT attack, the attacker is required to have access to the functional IC along with the obfuscated netlist, and different SAT-hard schemes, such as Yasin et al. (2016a) and Xie and Srivastava (2018), are proposed.

Furthermore, new obfuscation schemes that focus on non-Boolean behavior of circuits (Xie and Srivastava, 2017), which are not convertible to an SAT circuit, is proposed for SAT resilience. Some of such defenses include adding cycles into the design (Roshanisefat et al., 2018). Adding cycles into the design may cause the SAT attack to get stuck in an infinite loop. However, advanced SAT-based attacks, such as cycSAT (Zhou et al., 2017), can extract the correct key despite employing such defenses. Before the proposed defense ensures robustness against SAT attacks, the defenders need to run the rigorous simulations, which could range from few minutes up to years.

### 4.2. Graph Neural Networks

Many graphs and geometric convolution methods have been proposed recently for modeling graph data (Bronstein et al., 2017; Hamilton et al., 2017b; Zhang et al., 2018; Zhou et al., 2018; Wu et al., 2019). The spectral convolution methods (Defferrard et al., 2016; Kipf and Welling, 2017) are the mainstream algorithms developed as the graph convolution methods. The theory of these methods is based on the graph Fourier analysis (Shuman et al., 2013). The polynomial approximation was first proposed by Hammond et al. (2011). Inspired by this, graph convolutional neural networks (GCNNs) (Defferrard et al., 2016) successfully generalize the powerful convolutional neural networks (CNNs) in dealing with Euclidean data to modeling graph-structured data. Kipf and Welling (2017) proposed a simplified type of GCNNs, called graph convolutional networks (GCNs). The GCN model naturally integrates the connectivity patterns, features attributes of graph-structured data, and outperforms many state-of-the-art methods.

## 5. Evaluation

### 5.1. Benchmark Datasets

Experiments were conducted on a machine with Intel (R) Xeon (R) CPU E3-2136 with 32 GB memory^1^. Evaluation instances were extracted from ISCAS-89 benchmarks^2^, and each instance is encoded as 3-CNF-SAT (three literals in one clause) by Tseytin transformation. An 80/20% split was employed for training/testing set. These benchmarks were synthesized using the Synopsys DC Compiler with the help of 32/28 nm Generic library^3^. The synthesized netlist was further converted to the bench format as per the SAT-solver requirements (Subramanyan et al., 2015). The in-house developed script replaces the gates with the lookup table of size 2, as described in a study by Kamali et al. (2018). The output of the script is the obfuscated bench file along with the adjacency matrix. The obfuscated bench file along with the original benchmark were given to the SAT-solver as input parameters, and a modern SAT-solver (MiniSAT-based) implementation (Subramanyan et al., 2015) was used to get the runtime to estimate.

### 5.2. Baselines and Metrics

We compare, against several state-of-the-art regression models^4^: Linear Regression (LR), Passive Aggressive Regression (PAR), LASSO, Support Vector Regression (SVR), Ridge Regression, Orthogonal Matching Pursuit (OMP), SGD Regression, Least Angle Regression (LARS), Theil-Sen Estimators (Theil), and Multilayer Perceptron (MLP). These regression models cannot directly learn pattern on graph data, so the experiments prepared several predefined features, such as *size of the clause, size of the variable, the ratio of clause size to variable size*, and so on (i.e., feature 1–27 of Figure 2 in Devlin and O'Sullivan, 2008). From the state-of-art graph deep learning models, competitive GNN models, such as GCN (Kipf and Welling, 2017), GAT (Veličković et al., 2017), GraphSAGE (Hamilton et al., 2017a), were selected as another set of baselines. Since GCN/GAT/GraphSAGE were designed for node embeddings originally, we took mean function to aggregate all node representations in each single CNF graph instance and added three fully-connected layers to predict runtime. Another competitive baseline is encoding SAT as images and applying CNNs (DLAP) (Loreggia et al., 2016). In this experiment, the mean square error (MSE) of the runtime prediction task was utilized as the metric for performance evaluation. All experiments were repeated five times, and the average of metrics was shown.

### 5.3. Runtime Prediction Task

**Figure 4:**
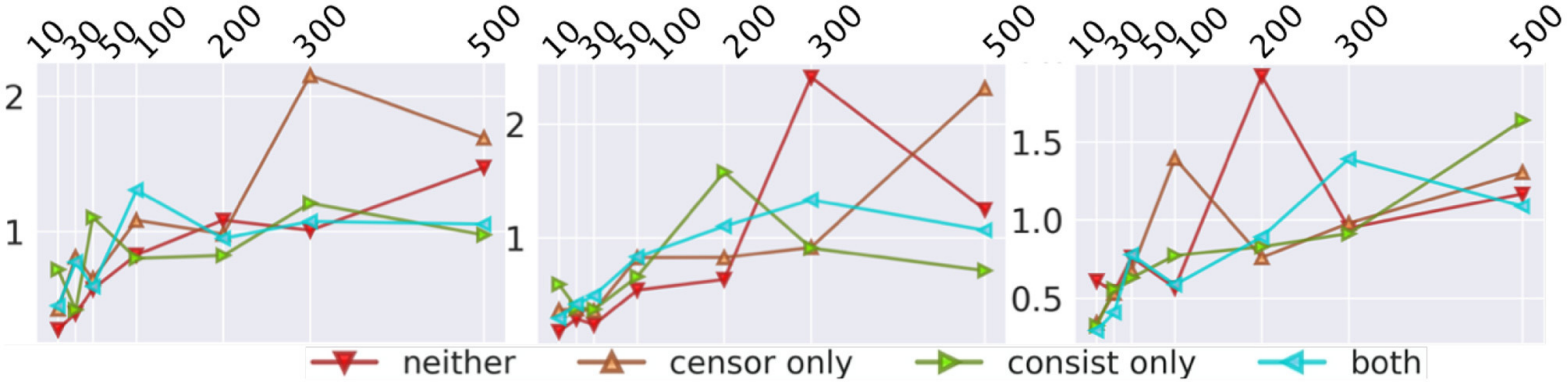
Performance Comparison on Benchmark. X-axis is the threshold (in seconds) for censoring the datasets, while y-axis indicates mean square error (MSE). Left to right are c432, c499, c880 datasets.

Figure 4 shows the performance comparison among baselines and the proposed method. There were four groups in the first subfigure: 1st group, as highlighted, contains most normal regression models; 2nd group, as marked, contains graph neural network models, and the remaining two are SGD (top and purple) and DSAG (bottom and red). It is obvious that normal regression, such as linear model, can hardly characterize non-linearity. Introducing graph topology, the 2nd group improves the normal regression models significantly (Note that the y-axis is in log scale). Further, the proposed DSAG improves the 2nd group dramatically. Several regression models in the 1st group have higher errors in smaller thresholds (e.g., 10 and 30), which is because a smaller threshold keeps less data for regression. On the contrary, the error of DSAG decreases significantly since DSAG still can utilize the censored data. This superiority of DSAG is consistent throughout all three datasets. Detailed numbers of runtime prediction is provided in Tables 1–3.

**Table 1:** Performance comparison on c432.

	**10**	**30**	**50**	**100**	**200**	**300**	**500**
Linear	3.1612e+00	2.1793e+01	6.3305e+01	1.1966e+02	5.5299e+02	1.0539e+03	2.5017e+03
Ridge	3.8178e+00	2.7043e+01	7.6082e+01	1.3196e+02	7.7698e+02	1.1116e+03	2.9605e+03
Lasso	4.5429e+00	3.0166e+01	8.6493e+01	1.4276e+02	1.1425e+03	1.6504e+03	3.1290e+03
MLP	1.2003e+02	4.0424e+02	1.6597e+02	1.9531e+02	1.5453e+03	2.8539e+03	4.3616e+03
Lars	6.1069e+04	1.9969e+07	1.8832e+02	9.9658e+02	3.5244e+03	1.4317e+10	2.2377e+04
OMP	3.3470e+00	3.0490e+01	8.7874e+01	1.4623e+02	6.0280e+02	1.2861e+03	3.0199e+03
SGD	6.0536e+30	2.8940e+30	1.8046e+31	1.3786e+31	1.1936e+31	4.9351e+30	8.8286e+30
PAR	1.7315e+01	4.5457e+01	1.1420e+02	2.6134e+02	1.7128e+03	3.8690e+03	7.4445e+03
Theil	4.0361e+00	3.0620e+01	1.1132e+02	1.8472e+02	1.0692e+03	1.1447e+03	3.2790e+03
SVR	5.8683e+00	4.0667e+01	1.1367e+02	1.5026e+02	1.4733e+03	2.3860e+03	3.4118e+03
DLAP	3.6971e+00	2.5654e+01	8.3923e+01	1.5056e+02	8.8146e+02	2.3792e+03	2.9953e+03
GAT	1.1647e+04	1.8566e+00	2.9676e+00	2.7502e+00	8.0696e+00	1.0336e+01	1.6019e+01
GAT-SA	9.1760e+00	5.4492e+01	1.3112e+02	3.1440e+02	7.3426e+02	4.5563e+02	8.9465e+02
GCN	1.1147e+04	1.8642e+00	3.0435e+00	2.9381e+00	1.0127e+01	1.0484e+01	1.6038e+01
GCN-SA	9.0540e+00	5.3624e+01	1.3155e+02	3.1420e+02	6.4244e+02	4.7414e+03	8.9572e+03
Sage	1.2215e+04	1.8586e+00	2.9289e+00	3.1756e+00	7.8375e+00	1.1503e+01	1.5929e+01
Sage-SA	9.0048e+00	5.8747e+01	1.3238e+02	3.1578e+02	7.9653e+02	4.6765e+02	9.0187e+03
DSAG-WB	1.6674e+06	1.1813e+02	8.8919e+04	5.0438e+06	6.4014e+07	6.8864e+09	1.5945e+08
DSAG	3.4140e-01	5.0372e-01	1.1775e+00	7.9674e-01	1.4410e+00	3.0248e+00	1.9715e+00

**Table 2:** Performance comparison on c499.

	**10**	**30**	**50**	**100**	**200**	**300**	**500**
Linear	1.1325e+00	7.2451e+00	2.3523e+01	2.0986e+02	3.6043e+02	9.9268e+02	4.9533e+02
Ridge	1.0289e+00	8.5923e+00	2.2871e+01	2.1248e+02	4.2973e+02	1.0718e+03	5.1005e+02
Lasso	1.5789e+00	1.1583e+01	3.2713e+01	2.5499e+02	4.7528e+02	1.1959e+03	5.5367e+02
MLP	2.5265e+00	1.8087e+01	4.6606e+01	3.2670e+02	6.9622e+02	1.5558e+03	8.1077e+02
Lars	9.6250e+00	5.4189e+01	2.3605e+01	2.1020e+02	3.6203e+02	1.8526e+03	5.7809e+03
OMP	9.9897e-01	9.7863e+00	2.9870e+01	2.3973e+02	4.3046e+02	1.1364e+03	5.7320e+02
SGD	7.0854e+30	7.8799e+30	9.1582e+30	1.8021e+31	5.7861e+30	6.6850e+30	1.0180e+31
PAR	3.8185e+00	3.7536e+01	4.8887e+01	3.3999e+02	6.2005e+02	1.5992e+03	8.1596e+02
Theil	1.3708e+00	1.2147e+01	3.1260e+01	2.7528e+02	4.7761e+02	1.3549e+03	5.3149e+02
SVR	4.4410e+00	6.6629e+01	7.0891e+01	3.6558e+02	6.5791e+02	1.6024e+03	7.0729e+02
DLAP	2.7471e+00	1.6825e+01	3.7197e+01	2.8338e+02	5.6832e+02	1.4428e+03	6.3257e+02
GAT	2.2144e+03	1.6008e+00	2.6829e+00	3.9028e+00	4.0831e+00	6.3761e+00	3.7974e+00
GAT-SA	7.7677e+00	4.4693e+01	7.0322e+01	2.1557e+02	6.9997e+02	1.3770e+02	1.6765e+02
GCN	7.5068e+03	1.5998e+00	2.7845e+00	3.9008e+00	4.0753e+00	6.3694e+00	3.8145e+00
GCN-SA	7.7510e+00	4.3631e+01	7.1336e+01	1.9336e+02	7.1150e+02	1.3481e+03	1.6710e+03
Sage	3.2712e+03	1.5777e+00	2.6410e+00	4.4357e+00	4.1685e+00	6.0335e+00	3.8338e+00
Sage-SA	7.7442e+00	4.3321e+01	7.1199e+01	1.8746e+02	6.9669e+00	1.3486e+03	1.6615e+03
DSAG-WB	2.7857e+15	5.8248e+16	1.2390e+15	1.0059+16	1.7777e+09.0	3.9907e+15	4.0283e+11
DSAG	3.4719e-01	5.2389e-01	6.4042e-01	1.3016e+00	2.0157e+00	2.7992e+00	1.9103e+00

**Table 3:** Performance comparison on c880.

	**10**	**30**	**50**	**100**	**200**	**300**	**500**
Linear	2.7754e+00	1.9417e+01	4.4460e+01	2.2991e+02	7.9404e+02	8.3623e+02	3.0158e+03
Ridge	3.8257e+00	2.6167e+01	6.3753e+01	2.6860e+02	8.8931e+02	9.9023e+02	3.3501e+03
Lasso	3.7568e+00	2.5577e+01	6.4954e+01	2.6742e+02	8.9272e+02	1.0711e+03	3.6088e+03
MLP	1.5144e+02	3.1003e+01	7.1854e+01	2.8338e+02	9.3473e+02	1.1511e+03	3.8307e+03
Lars	5.0124e+02	1.4411e+04	6.8009e+01	2.7046e+02	1.1785e+03	1.4192e+03	3.8229e+03
OMP	3.4928e+00	2.3846e+01	5.4433e+01	2.6425e+02	8.9263e+02	1.0718e+03	3.6112e+03
SGD	3.2375e+30	1.0856e+31	1.2071e+31	1.0439e+31	1.0808e+31	5.7292e+30	3.1060e+30
PAR	7.1275e+00	4.1192e+01	1.2508e+02	3.0963e+02	1.0876e+03	1.3523e+03	4.5832e+03
Theil	3.3507e+00	2.0203e+01	5.3617e+01	2.5220e+02	8.9015e+02	1.0767e+03	3.8655e+03
SVR	4.5527e+00	3.0250e+01	7.3403e+01	3.1302e+02	1.0250e+03	1.2775e+03	4.3970e+03
DLAP	4.1121e+00	2.2928e+01	6.3833e+01	1.8452e+02	1.0289e+03	1.2631e+03	1.2631e+03
GAT	6.0733e+02	1.7071e+00	2.4918e+00	3.8402e+00	5.1399e+00	5.5019e+00	8.8471e+00
GAT-SA	7.5801e+00	4.0828e+01	8.1052e+01	2.2951e+02	8.3567e+02	1.4445e+03	3.6633e+03
GCN	6.1568e+02	1.7124e+00	2.4739e+00	3.8181e+00	5.1608e+00	5.4951e+00	8.8469e+00
GCN-SA	7.3204e+00	4.0817e+01	8.1533e+01	2.2746e+02	8.2630e+02	1.3515e+03	3.5722e+02
Sage	6.3035e+02	1.6968e+00	2.5505e+00	3.8779e+00	5.1838e+00	5.3649e+00	8.8540e+00
Sage-SA	7.2826e+00	4.1120e+01	8.1561e+01	2.3649e+02	8.3934e+02	1.3618e+03	3.6242e+03
DSAG-WB	3.4867e+01	4.0870e+04	5.4838e+05	8.6439e+11	1.0066e+07	2.0078e+06	7.9632e+05
DSAG	5.6682e-01	1.1624e+00	8.1095e-01	2.6881e+00	1.5796e+00	1.9264e+00	1.8682e+00

### 5.4. Ablation Test

**Figure 5:**
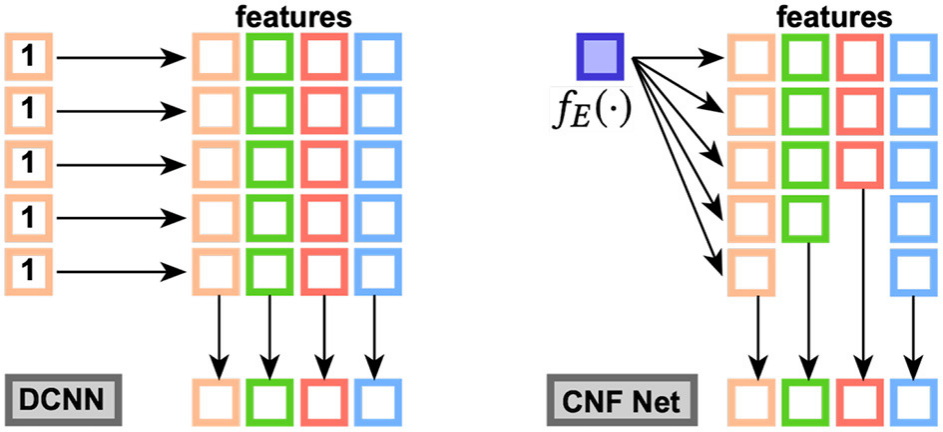
Ablation Test for DSAG: *neither* means no censor and consistence loss, while *both* indicates using both censor and consistence loss. Left to right are c432, c499, c880 datasets.

To study each component in survival analysis, an ablation test on the proposed three losses was carried out. Figure 5 shows the performance using different loss combinations. If using neither censor loss or consistence loss, the model only applies MSE as the only objective and is inclined to the output with bigger errors, such as the threshold of 300 in the second dataset and threshold of 200 in the third dataset. If using only censor loss, the model also has large errors, such as threshold of 300 in the first dataset, threshold of 500 in the second, and threshold of 100 in the third. The remaining two combinations, i.e., consistence loss only and *both*, have similar performance and outperform the other two (i.e., *neither* and *censor only*). *Both* includes the censor loss and consistence loss, but the consistence loss performed better than censor loss. These two losses both compare the label and the representation of the censored data, but the difference is that **censor loss** use a classification model with cross-entropy, while *consistence loss* is associated with regression component. Therefore, it is natural that *consistence loss* is better than *censor loss* since regression model has loss value with fine granularity. Combining *consistence loss* and *censor loss, both* achieves the level of the better one, i.e., *consistence loss*. Therefore, we can conclude that *consistence loss* is the key factor that improves the one without survival analysis (i.e., *neither* in red). Detailed numbers of ablation test is provided in Tables 4–6.

**Table 4:** Ablation test on c432.

	**10**	**30**	**50**	**100**	**200**	**300**	**500**
Neither	8.3943e-01	7.1515e-01	1.1436e+00	7.6344e-01	5.8402e+00	1.5851e+00	2.2145e+00
Censor	4.0860e-01	7.0290e-01	9.6342e-01	3.0432e+00	1.1402e+00	1.6648e+00	2.6891e+00
Consist	3.8342e-01	7.4995e-01	8.7840e-01	1.1675e+00	1.2938e+00	1.4941e+00	4.1502e+00
Both	3.4140e-01	5.0372e-01	1.1775e+00	7.9674e-01	1.4410e+00	3.0248e+00	1.9715e+00

**Table 5:** Ablation test on c499.

	**10**	**30**	**50**	**100**	**200**	**300**	**500**
Neither	1.9976e-01	3.3945e-01	2.7204e-01	7.2224e-01	8.9144e-01	1.0160e+01	2.4844e+00
Censor	4.4624e-01	4.5780e-01	4.3349e-01	1.2885e+00	1.2926e+00	1.5043e+00	9.1251e+00
Consist	8.0903e-01	4.7088e-01	4.5495e-01	9.3713e-01	3.8482e+00	1.4911e+00	1.0419e+00
Both	3.4719e-01	5.2389e-01	6.4042e-01	1.3016e+00	2.0157e+00	2.7992e+00	1.9103e+00

**Table 6:** Ablation test on c880.

	**10**	**30**	**50**	**100**	**200**	**300**	**500**
Neither	3.1560e-01	4.8206e-01	7.7573e-01	1.2841e+00	1.9549e+00	1.7395e+00	3.3581e+00
Censor	5.2762e-01	1.2513e+00	9.1296e-01	1.9529e+00	1.6656e+00	7.6368e+00	4.4233e+00
Consist	1.0473e+00	5.2228e-01	2.0164e+00	1.2262e+00	1.2743e+00	2.3473e+00	1.6471e+00
Both	5.6682e-01	1.1624e+00	8.1095e-01	2.6881e+00	1.5796e+00	1.9264e+00	1.8682e+00

The default setting for the cumulative death distribution function (i.e., 1 − *S* where *S* is the survival function) is the exponential function, which is not only simple but also effective. We also conducted different distribution assumptions, which include log-logistic and Weibull (DSAG-WB). Due to the inversion in log-logistic (e.g., a small value will cause a huge value), the gradient is unstable and failed to learn a model. This is because Weibull set a higher-order on the scale (*e*^*t*^) regarding the time, which results in a much higher result and error, so Weibull may not be suitable for our problem.

### 5.5. Training and Prediction Efficiency

In c432, each training epoch takes 30.65 s, while c499 takes 36.60 s, and c880 takes 37.32 s. The training process takes around 25 epochs to achieve convergence for all the datasets. Therefore, it takes 766.25 s for c432, 915 s for c499, and 933 s for c800. Theoretically, the exponential time hypothesis (Impagliazzo and Paturi, 1999) states that no algorithm can solve 3-SAT in exp(*o*(*n*)) time, where the modern SAT solver follows exponential runtime growth as the best case w.r.t. the number of variables. On the contrary, DSAG is a neural network model, and neural networks accept the intermediate feature with fixed dimensions as input and apply vector multiplication with parameters. Therefore, DSAG only needs constant complexity of O(1) to predict. Therefore, DSAG is exponentially faster than actually running the SAT solver in the runtime estimation task. This significantly boosts runtime estimation, especially when the SAT solver runtime is large. Specifically, DSAG takes 0.9223 s for c432 average, 1.0188 s for c499, and 1.1152 s for c880.

## 6. Conclusion

This study presents a novel framework to estimate the runtime of circuit deobfuscation. There is no existing study on representation learning to address the critical challenges, including learning on dynamic size, logic operation of the CNF-SAT instance, and utilizing censored data. We proposed an energy-based kernel that is designed to aggregate features of the varying-size graph. By introducing survival analysis, DSAG integrates the information from the censored data and improves the overall performance. Experiments on benchmarks demonstrated the advantageous performance of the proposed model over the existing graph neural networks and normal regression models.

## Data Availability Statement

The datasets presented in this study can be found in online repositories. The names of the repository/repositories and accession number(s) can be found at: https://github.com/demovis/DSAG.

## Author Contributions

ZC and LZhan designed the model and conducted the experiments. LZhao provided the feedback on the idea, writing, and connected with all co-authors. GK, HK, SR, and SP provided the problem definition, contributed the domain knowledge, and verified our task. HH and C-TL offered the feedback on the proposed method and writing. All authors contributed to the article and approved the submitted version.

## Conflict of Interest

The authors declare that the research was conducted in the absence of any commercial or financial relationships that could be construed as a potential conflict of interest.

## Footnotes

^1^Datasets and codes are available at https://github.com/demovis/DSAG.

^2^http://www.pld.ttu.ee/~maksim/benchmarks/iscas85/verilog/

^3^https://www.synopsys.com

^4^https://scikit-learn.org/stable/modules/linear_model.html
